# CAA Accumulation in the Tg2576 APPSw Mouse Model Is Associated With Inflammation and Vascular Remodeling

**DOI:** 10.1093/geroni/igaf122.1007

**Published:** 2025-12-31

**Authors:** Katelynn Krick, Donna Wilcock

**Affiliations:** Indiana University School of Medicine, Indianapolis, Indiana, United States; Indiana University, Indianapolis, Indiana, United States

## Abstract

Cerebral amyloid angiopathy (CAA) is an extremely common pathology of Alzheimer’s disease (AD) included under vascular contributions to cognitive impairment and dementia (VCID). CAA has been reported in 78-98% of AD cases and has clinical significance when considering amyloid related imaging abnormalities (ARIA) that arise when using amyloid targeting immunotherapies. Despite its prevalence, studies addressing CAA mechanisms have been scarce and there are clear gaps in our understanding of how CAA progresses. This study uses Tg2576 mice, who develop CAA over time, to establish a time course of CAA progression at 8-, 14-, and 20-months of age. We identify changes in transcriptomic signatures of glial cells using NanoString nCounter and targeted protein changes using Nanostring Digital Spatial Profiling. Meso Scale Discovery and immunohistochemistry are used to establish disease progression. In this study, we saw many changes primarily associated with inflammatory response, with some changes being transient (Tnf, Lsr; VEGF) and others remaining chronically altered (Osmr, Ccl3; CTSD). Overarchingly, many of these changes relate to the perpetuation of inflammation or recruiting additional immune support, which we see in across our timepoints. Further, we identified differences in abundance of proteins (CD45, GFAP, CD31) based on presence of CAA positive vessels within a brain region. We also identified sex-specific differences in CAA burden, as well as how glial reactivity and vessel density change during disease progression. This data represents a comprehensive analysis of CAA progression and differential responses to parenchymal and vascular amyloid that could inform future basic and clinical studies.

